# Barriers to accessing healthcare among women in Ghana: a multilevel modelling

**DOI:** 10.1186/s12889-020-10017-8

**Published:** 2020-12-17

**Authors:** Abdul-Aziz Seidu, Eugene Kofuor Maafo Darteh, Ebenezer Agbaglo, Louis Kobina Dadzie, Bright Opoku Ahinkorah, Edward Kwabena Ameyaw, Justice Kanor Tetteh, Linus Baatiema, Sanni Yaya

**Affiliations:** 1 Department of Population and Health, University of Cape Coast, Cape Coast, Ghana; 2 College of Public Health, Medical and Veterinary Sciences, James Cook University, Townsville, Queensland Australia; 3 Department of English, University of Cape Coast, Cape Coast, Ghana; 4 The Australian Centre for Public and Population Health Research (ACPPHR), Faculty of Health, University of Technology Sydney, Sydney, Australia; 5 School of International Development and Global Studies, University of Ottawa, Ottawa, Canada; 6 The George Institute for Global Health, The University of Oxford, Oxford, UK

**Keywords:** Barriers, Ghana, Women’s health, Multi-level analysis, DHS, Public health, Global health

## Abstract

**Background:**

Women’s health remains a global public health concern, as enshrined in the Sustainable Development Goals. This study, therefore, sought to assess the individual and contextual factors associated with barriers to accessing healthcare among women in Ghana.

**Methods:**

The study was conducted among 9370 women aged 15–49, using data from the 2014 Ghana Demographic and Health Survey. Barrier to healthcare, derived from four questions— whether a woman faced problems in getting money, distance, companionship, and permission to see a doctor—was the outcome variable. Descriptive and multilevel logistic regression analyses were carried out. The fixed effect results of the multilevel logistic regression analyses were reported using adjusted odds ratios at a 95% confidence interval.

**Results:**

More than half (51%) of the women reported to have at least one form of barrier to accessing healthcare. Women aged 45–49 (AOR = 0.65, CI: 0.49–0.86), married women (AOR = 0.71, CI:0.58–0.87), those with a higher level of education (AOR = 0.51, CI: 0.37–0.69), those engaged in clerical or sales occupation (AOR = 0.855, CI: 0.74–0.99), and those who were covered by health insurance (AOR = 0.59, CI: 0.53–0.66) had lower odds of facing barriers in accessing healthcare. Similarly, those who listened to radio at least once in a week (AOR =0.77, CI: 0.66–0.90), those who watched television at least once a week (AOR = 0.75, CI: 0.64–0.87), and women in the richest wealth quintile (AOR = 0.47, CI: 0.35–0.63) had lower odds of facing barriers in accessing healthcare. However, women who were widowed (AOR = 1.47, CI: 1.03–2.10), those in the Volta Region (AOR 2.20, CI: I.38–3.53), and those in the Upper West Region (AOR =2.22, CI: 1.32–3.74) had the highest odds of facing barriers to healthcare accessibility.

**Conclusion:**

This study shows that individual and contextual factors are significant in predicting barriers in healthcare access in Ghana. The factors identified include age, marital status, employment, health insurance coverage, frequency of listening to radio, frequency of watching television, wealth status, and region of residence. These findings highlight the need to pay critical attention to these factors in order to achieve the Sustainable Development Goals 3.1, 3.7, and 3.8. It is equally important to strengthen existing strategies to mitigate barriers to accessing healthcare among women in Ghana.

**Supplementary Information:**

The online version contains supplementary material available at 10.1186/s12889-020-10017-8.

## Background

Women’s health remains a global public health concern, as the health and wealth of any society largely depend on the health and wealth of its women [1]. The health of women is fundamental to socioeconomic development, particularly in Africa [2]. Women’s health was emphasized by the fourth World Conference on Women held in Beijing in 1995 [3]. The Sustainable Development Goals (SDGs) also pay special attention to women’s health. For example, targets 3.8 and 3.7 of the SDG-3 emphasize universal health coverage and access to sexual and reproductive health services, including family planning information and education, and integrating reproductive health into national strategies and plans by 2030 [4, 5]. Specifically, the SDGs aim to further reduce the maternal mortality rate, which is evident in Goal 3.1 (to reduce the global maternal mortality rate to less than 70 per 100,000 live births by 2030).

Though these targets have resulted in some improvement in women’s health, there is still more to be done. In 2016, 303,000 women over the world lost their lives to maternal mortality [1, 5] mainly due to preventable diseases, with sub-Saharan Africa being disproportionately affected. Besides, in almost all the countries of the world, a lot of women have died, with a lot disabled in low- and middle-income countries, as a result of non-communicable diseases [6]. African women are also undernourished, and the burden of HIV/AIDS is heavier, with related morbidity and mortality accounting for 89% of global women’s disability-adjusted life years (DALYs) [2]. Studies have shown that women do not get medical care when they need it, and they do not get the best care, which leads to poor health. In addition, studies in Ethiopia [7, 8], Rwanda [9], Cameroon, and India [10] indicate that individual and contextual factors may hinder women’s access to medical services. Women in sub-Saharan Africa also face some barriers that prevent them from making effective reproductive health decisions [11, 12].

Over the years, the government of Ghana has attempted to improve access to health services through some policies, with the most current one being the National Health Insurance Scheme (NHIS). Generally, the policy allows all individuals registered to have free access to healthcare. The NHIS has particularly been important to women, as it allows all pregnant women under the scheme to have free access to maternal healthcare services, including antenatal care, delivery services, postnatal care, and neonatal care. Notwithstanding, a study by Penfold [13] found that the policy comes with other financial and logistical barriers that limit access for a lot of women. It must be noted that Penfold’s [13] study is not only older than a decade but also limited its focus to only Volta and Central Regions, which questions its generalizability to the entire nation. The focus of Kumi-Kyereme, Amu, and Darteh [14] was also very narrow. They found that long queues and waiting times, poor quality of medicines, and negative attitudes of service providers are obstacles to access to healthcare in Cape Coast, Ghana. In the present study, we used nationally representative data to investigate the individual and contextual factors associated with barriers to access healthcare among women in Ghana. Focusing on women in this study is needful as the findings could help reduce barriers women face in accessing healthcare. This could also go a long way to reduce maternal mortality cases which is currently 310/100,000 in Ghana [15] and help in the attainment of the SDGs 3.1 of reducing maternal mortality to 70/100,000 live births by 2030.

## Materials and methods

### Data source

The data used for this study forms part of the 2014 Ghana Demographic and Health Survey (GDHS). The survey adopted a two-stage stratified sampling technique. Before the sampling, the regions in the country were apportioned into urban and rural areas. A two-stage sampling procedure was used to sample units (clusters) consisting of enumeration areas (EAs). The first stage involved selecting sample points (clusters) consisting of EAs ﻿delineated for the 2010 Population and Housing Census. A total of 427 clusters were selected, 216 in urban areas and 211 in rural areas. The second stage saw the systematic selection of 30 households from each cluster through probability sampling, and this yielded a total of 12,831 households. For this study, we focused on 9370 women of reproductive age (15-49) who had complete information on the variables the present study was interested in. Details of the methodology, pretesting, training of field workers, the sampling design, and selection are available in the GDHS final report [16] which is also available online at https://dhsprogram.com/publications/publication-fr307-dhs-final-reports.cfm?cssearch=93962_1. We relied on the “Strengthening the Reporting of Observational Studies in Epidemiology” (STROBE) statement in conducting this study and writing the manuscript.

### Variables

#### Outcome variable

The outcome variable was barrier to healthcare accessibility. In the GDHS, each woman was interviewed to respond to four questions on barriers to healthcare access based on obtaining money, distance to a health facility, getting permission for treatment, and not wanting to go alone. If a woman faced at least one or more of the problems (money, distance, companionship, and permission), she is considered to have a barrier to healthcare access and coded as “1”, whereas if she didn’t report money, distance, companionship, and permission-related barriers, she is considered not to have a barrier of healthcare access and coded as “0” [9, 17, 18].

#### Independent variables

Individual and contextual (household and community-level factors) were considered as independent variables in this study. The individual-level factors included age, marital status, educational level, ethnicity, employment, religion, parity, health insurance subscription, and exposure to mass media (radio, newspaper and television). The contextual level variables included in the study are sex of household head, household wealth status, residence, region and neighborhood socio-economic status. The community-level socio-economic variable was generated by aggregating the individual-level data into cluster, except for place of residence and geographical region that were taken as they are. Neighbourhood socioeconomic disadvantage was operationalized with a principal component comprising the proportion of respondents with no formal education, unemployed, rural resident, and living below the poverty level (asset index below 20% poorest quintile). A standardized score with mean 0 and standard deviation 1 was generated from this index, with higher scores being indicative of the lower socioeconomic status (SES). We divided the resultant scores into tertiles to allow for nonlinear effects and provided results that were more readily interpretable in the policy arena [19].

### Statistical analysis

The data were analyzed with Stata version 14.2 for macOS. Three basic steps were followed to analyze the data. The first step was the use of descriptive statistics to describe the sample and also crosstab all the independent variables against each barrier to healthcare access and at least one barrier. The second step was a bivariate analysis to select potential variables for the regression analysis. Variables that were statistically significant in bivariate analyses at the α = 0.05, were retained for a multilevel analysis. The multilevel analysis was made up of two levels and assessed the individual and contextual factors associated with barriers to healthcare access. Clusters were considered as a random effect to account for the unexplained variability at the community level [20, 21]. We fitted four models. Firstly, we fitted the empty model, Model I that had no predictors (random intercept). Afterwards, the Model II contained only the individual-level variables, Model III with only contextual level variables, and Model IV, both individual-level and contextual level variables. For all models, we presented the adjusted odds ratio and associated 95% confidence intervals. These models were fitted by a Stata command “melogit” for the identification of predictors with the outcome variable. For model comparison, we used the log-likelihood ratio (LLR) and Akaike Information Criteria (AIC) test. The highest log-likelihood and the lowest AIC wins the best fit model. Using the variance inflation factor (VIF), the multicollinearity test showed that there was no evidence of collinearity among the independent variables (Mean VIF = 1.9, Maximum VIF = 4.4 and Minimum VIF = 1.0). Sample weight (v005/1,000,000) was applied in all the analysis to correct for over- and under-sampling while we used the SVY command to account for the complex survey design and generalizability of the findings.

## Results

**Fig. 1:**
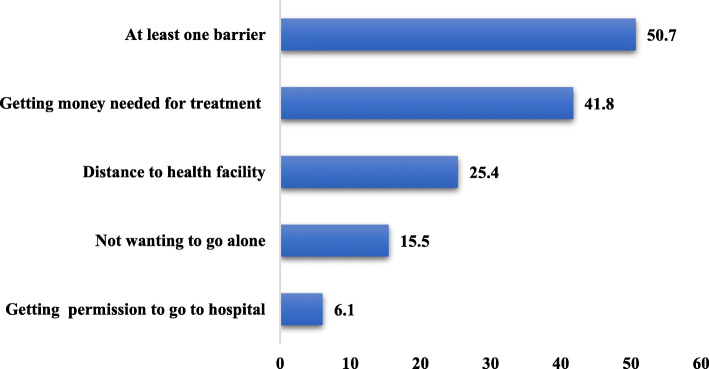
Barriers in access to healthcare among women. Source: 2014 GDHS

As shown in Fig. 1, more than half (51%) of the women reported to have at least one form of barrier to accessing healthcare. About 42% of the women reported that getting money for treatment was a barrier in accessing healthcare. Also, 25% complained of distance to health facility as a barrier while 16% mentioned not wanting to go alone and 6% also indicated that they needed permission each time before they could access healthcare.

### Socio-demographic characteristics and barriers to healthcare access among women in Ghana

Table 1 shows the socio-demographic characteristics and barriers to healthcare access among women in Ghana. About 80% of the respondents professed to be Christians. Also, about 54% were rural dwellers. The majority of the women aged 15–19 (59%) had at least one barrier to healthcare accessibility, as compared with the other age groups in the study. With marital status, 61.6% of widowed women indicated they have faced at least one barrier to healthcare. About 63.5% of those with no education, 61.6% of those in other ethnic groups, 67.0% of those in agriculture, 78.2% of those who professed the traditional religion, 58.1% of those with parity 4 or more, and 57.6% of those who were subscribed to health insurance indicated they face at least one barrier to healthcare.

**Table 1:** Background characteristics and barriers to healthcare access among women in Ghana (*n* = 9370)

Variable	***N*** = 9370		Permission	Money	Distance	Alone	At least one barrier
	Weighted Frequency	Weighted Percentage	n (%)	n (%)	n (%)	n (%)	
Age			*p* < 0.001	*p* < 0.001	*p* = 0.001	*p* < 0.001	*p* < 0.001
15–19	1622	17.3	141 (8.7)	755 (46.6)	452 (27.9)	397 (24.5)	58.5
20–24	1609	17.2	111 (6.9)	654 (40.7)	406 (25.3)	238 (14.8)	51.2
25–29	1599	17.1	73 (4.6)	540 (33.8)	335 (20.9)	204 (12.8)	43.6
30–34	1367	14.6	72 (5.3)	543 (39.7)	341 (25)	181 (13.2)	46.5
35–39	1289	13.8	76 (5.9)	533 (41.4)	313 (24.3)	169 (13.1)	48.3
40–44	1029	11.0	54 (5.3)	489 (47.5)	279 (27.1)	142 (13.8)	55.2
45–49	855	9.1	39 (4.6)	397 (46.4)	249 (29.1)	123 (14.4)	53.5
**Marital status**			*p* < 0.001	*p* < 0.001	*p* = 0.004	*p* < 0.001	*p* < 0.001
Never married	3088	33.0	235 (7.6)	1271 (41.2)	731 (23.7)	581 (18.8)	52.0
Married	3952	42.2	199 (5)	1552 (39.3)	1047 (26.5)	572 (14.5)	47.6
Cohabitation	1351	14.4	92 (6.8)	579 (42.8)	362 (26.8)	172 (12.8)	52.0
Widowed	252	2.7	11 (4.6)	138 (54.8)	68 (27)	45 (17.7)	61.6
Divorced	726	7.8	30 (4.1)	372 (51.2)	167 (23)	84 (11.5)	55.9
**Educational level**			*p* = 0.002	*p* < 0.001	*p* < 0.001	*p* < 0.001	*p* < 0.001
No education	1788	19.1	123 (6.9)	981 (54.9)	675 (37.7)	376 (21)	63.5
Primary	1667	17.8	113 (6.8)	849 (50.9)	482 (28.9)	254 (15.3)	58.7
Secondary	5322	56.8	310 (5.8)	1992 (37.4)	1158 (21.8)	767 (14.4)	46.8
Higher	592	6.3	21 (3.6)	90 (15.1)	61 (10.4)	57 (9.6)	24.9
**Ethnicity**			*p* < 0.001	*p* < 0.001	*p* < 0.001	*p* < 0.001	*p* < 0.001
Akan	4695	50.1	285 (6.1)	1694 (36.1)	978 (20.8)	591 (12.6)	45.0
Ga Adangme/ewe	1989	21.2	81 (4.1)	851 (42.8)	512 (25.8)	328 (16.5)	53.6
Mole -dagbani	1385	14.8	88 (6.4)	659 (47.6)	444 (32.1)	242 (17.5)	55.8
Other	1302	13.9	112 (8.6)	708 (54.4)	441 (33.9)	294 (22.6)	61.6
**Employment**			*p* < 0.001	*p* < 0.001	*p* < 0.001	*p* < 0.001	*p* < 0.001
Not working	2195	23.4	180 (8.2)	956 (43.5)	558 (25.4)	443 (20.2)	53.9
Managerial	526	5.6	20 (3.8)	110 (20.9)	73 (13.9)	61 (11.7)	30.6
Clerical/sales	3552	37.9	179 (5)	1301 (36.6)	698 (19.6)	406 (11.4)	44.4
Agricultural	1750	18.7	127 (7.3)	1012 (57.8)	769 (43.9)	379 (21.7)	67.0
Services	198	2.1	3 (1.4)	60 (30.5)	30 (15.3)	12 (6)	37.7
Manual	1149	12.3	59 (5.1)	473 (41.2)	248 (21.6)	152 (13.3)	50.8
**Religion**			*p* < 0.001	*p* < 0.001	*p* < 0.001	*p* < 0.001	*p* < 0.001
Christianity	7516	80.2	451 (6)	3041 (40)	1833 (24)	1118 (15)	49.5
Islam	1415	15.1	78 (6)	589 (42)	370 (26)	232 (16)	51.3
Traditionalist	188	2.0	25 (13)	136 (72)	100 (53)	65 (35)	78.2
No religion	251	2.7	13 (5)	147 (59)	72 (29)	38 (15)	63.6
**Parity**			*p* < 0.001	*p* < 0.001	*p* < 0.001	*p* < 0.001	*p* < 0.001
0	2932	31.3	217 (7.4)	1128 (38.5)	690 (23.6)	566 (19.3)	50.3
1 to 3	3757	40.1	182 (4.9)	1415 (37.7)	818 (21.8)	478 (12.7)	45.7
4 or more	2681	28.6	167 (6.2)	1368 (51)	867 (32.4)	409 (15.3)	58.1
**Health insurance subscription**			*p* = 0.070	*p* < 0.001	*p* = 0.006	*p* = 0.070	*p* < 0.001
No	3526	37.6	233 (6.6)	1747 (49.5)	951 (27)	585 (16.6)	57.6
Yes	5844	62.4	334 (5.7)	2165 (37)	1424 (24.4)	869 (14.9)	46.6
**Frequency of listening to radio**			*p* = 0.001	*p* < 0.001	*p* < 0.001	*p* < 0.001	*p* < 0.001
Not at all	1469	15.7	103 (7)	799 (54.4)	479 (32.6)	312 (21.3)	62.6
less than once a week	3015	32.2	216 (7.2)	1348 (44.7)	807 (26.8)	462 (15.3)	53.1
At least once a week	4887	52.2	248 (5.1)	1764 (36.1)	1090 (22.3)	679 (13.9)	45.7
**Frequency of reading newspaper**			*p* = 0.407	*p* < 0.001	*p* < 0.001	*p* = 0.425	*p* < 0.001
Not at all	7603	81.1	476 (6.3)	3390 (44.6)	2073 (27.3)	1171 (15.4)	53.1
Less than once a week	953	10.2	52 (5.5)	309 (32.4)	180 (18.9)	171 (17.9)	43.9
At least once a week	814	8.7	39 (4.8)	213 (26.1)	123 (15.1)	112 (13.8)	36.4
**Frequency of watching television**			*p* < 0.001	*p* < 0.001	*p* < 0.001	*p* < 0.001	*p* < 0.001
Not at all	2203	23.5	151 (6.9)	1275 (57.9)	887 (40.3)	462 (21)	66.4
Less than once a week	2413	25.8	171 (7.1)	988 (41)	556 (23.1)	339 (14)	49.4
At least once a week	4754	50.7	245 (5.2)	1648 (34.7)	932 (19.6)	653 (13.7)	44.1
**Contextual factors**							
**Sex of head of household**			*p* = 0.39	*p* = 0.738	*p* < 0.001	*p* = 0.002	*p* = 0.106
Male	5742	61.3	352 (6.1)	2347 (40.9)	1613 (28.1)	942 (16.4)	50.8
Female	3628	38.7	215 (5.9)	1565 (43.1)	763 (21)	512 (14.1)	50.7
**Wealth**			*p* < 0.001	*p* < 0.001	*p* < 0.001	*p* < 0.001	*p* < 0.001
Poorest	1512	16.1	115 (7.6)	886 (58.6)	682 (45.1)	367 (24.3)	67.6
Poorer	1634	17.4	123 (7.5)	881 (54)	573 (35.1)	298 (18.3)	63.4
Middle	1934	20.6	128 (6.6)	883 (45.7)	458 (23.7)	265 (13.7)	53.9
Richer	2110	22.5	111 (5.2)	767 (36.3)	388 (18.4)	261 (12.4)	44.5
Richest	2181	23.3	91 (4.2)	494 (22.7)	275 (12.6)	263 (12)	32.7
**Neighbourhood socio-economic status**			*p* < 0.001	*p* < 0.001	*p* < 0.001	*p* < 0.001	*p* < 0.001
Low	3859	41.2	205 (5.3)	1274 (33)	629 (16.3)	515 (13.3)	42.1
Medium	3306	35.3	188 (5.7)	1388 (42)	768 (23.2)	415 (12.6)	50.5
High	2205	23.5	174 (7.9)	1250 (56.7)	979 (44.4)	524 (23.8)	66.2
**Residence**			*p* = 0.001	*p* < 0.001	*p* < 0.001	*p* < 0.001	*p* < 0.001
Urban	5030	53.7	280 (5.6)	1772 (35.2)	864 (17.2)	685 (13.6)	44.1
Rural	4340	46.3	287 (6.6)	2139 (49.3)	1511 (34.8)	769 (17.7)	58.4
**Religion**			*p* < 0.001	*p* < 0.001	*p* < 0.001	*p* < 0.001	*p* < 0.001
Western	1037	11.1	83 (8)	356 (34.3)	204 (19.7)	70 (6.8)	44.1
Central	934	10.0	39 (4.1)	320 (34.3)	170 (18.2)	111 (11.9)	42.3
Greater Accra	1891	20.2	70 (3.7)	470 (24.9)	238 (12.6)	252 (13.3)	35.4
Volta	719	7.7	16 (2.2)	445 (61.9)	232 (32.2)	121 (16.8)	69.8
Eastern	875	9.3	77 (8.8)	414 (47.4)	317 (36.3)	154 (17.6)	59.1
Ashanti	1791	19.1	123 (6.9)	883 (49.3)	474 (26.5)	309 (17.3)	55.9
Brong Ahafo	769	8.2	45 (5.8)	250 (32.5)	164 (21.3)	105 (13.6)	41.7
Northern	786	8.4	67 (8.6)	473 (60.2)	391 (49.8)	265 (33.7)	70.8
Upper East	358	3.8	16 (4.3)	165 (46)	72 (20)	34 (9.4)	51.5
Upper West	211	2.3	32 (15.3)	135 (63.7)	113 (53.3)	33 (15.4)	69.9

### Individual and contextual factors associated with barriers to healthcare access among women in Ghana

Tables 2 and 3 presents results of the fixed effects and random effects respectively on the multilevel logistic regression analysis of individual and contextual factors associated with barriers to healthcare concerning getting permission to go to hospital, getting money needed for treatment, distance to health facility, not wanting to go alone and at least one barrier. The results showed that women aged 40–44 had the lowest odd to have difficulty with wanting to go alone to the health facility [AOR = 0.547, CI = 0.38,0.78] and while women age 45–49 had the lowest odds of facing at least one barrier to healthcare accessibility (AOR = 0.65, CI: 0.49–0.86) compared to those aged 15–19.

**Table 2:** Multilevel logistic regression of individual and contextual factors associated with barriers to healthcare among women in Ghana (Fixed effects results)

Variable	Permission	Money	Distance	Alone	At least one barrier
	AOR [95%CI]	AOR [95%CI]	AOR [95%CI]	AOR [95%CI]	AOR [95%CI]
Age					
15–19	Ref	Ref	Ref	Ref	Ref
20–24	0.941 [0.70,1.27]	1.00 [0.73,1.05]	0.915 [0.75,1.12]	0.613***[0.49,0.77]	0.820^*^[0.68,0.98]
25–29	0.7 [0.51,1.11]	0.727** [0.58,0.91]	0.823 [0.64,1.05]	0.592***[0.45,0.78]	0.67^***^[0.54,0.83]
30–34	1.00 [0.56,1.33]	0.90 [0.69,1.12]	0.887 [0.67,1.17]	0.614**[0.45,0.84]	0.729^*^[0.57,0.93]
35–39	0.939 [0.60,1.48]	1.00 [0.65,1.09]	0.877 [0.66,1.17]	0.576**[0.41,0.80]	0.716^*^[0.55,0.93]
40–44	0.70 [0.42,1.13]	0.863 [0.66,1.14]	0.893 [0.66,1.21]	0.547***[0.38,0.78]	0.750^*^[0.57,0.99]
45–49	1.00 [0.40,1.15]	0.745*[0.56,0.99]	0.805 [0.59,1.11]	0.555** [0.39,0.80]	0.65^**^[0.49,0.86]
**Marital status**					
Never married	Ref	Ref	Ref	Ref	Ref
Married	0.70 [0.52,1.07]	0.604***[0.49,0.74]	0.854 [0.67,1.08]	1.05 [0.80,1.38]	0.71^**^[0.58,0.87]
Cohabitation	1.055 [0.72,1.54]	0.742** [0.60,0.92]	0.866 [0.67,1.11]	0.937 [0.70,1.25]	0.882 [0.71,1.10]
Widowed	0.738 [0.39,1.41]	1.378 [0.97,1.96]	0.976 [0.66,1.43]	1.498 [0.97,2.32]	1.469^*^[1.03,2.10]
Divorced	1.0 [0.42,1.12]	1.2 [0.91,1.53]	0.996 [0.74,1.34]	1.016 [0.71,1.45]	1.116 [0.86,1.44]
**Educational level**					
No education	Ref	Ref	Ref	Ref	Ref
Primary	1.1 [0.83,1.47]	1.007 [0.86,1.19]	0.914 [0.77,1.09]	0.835 [0.68,1.03]	0.903 [0.76,1.07]
Secondary	1.00 [0.67,1.19]	0.797**[0.68,0.93]	0.908 [0.76,1.08]	0.835 [0.68,1.03]	0.75^***^[0.64,0.89]
Higher	0.588 [0.31,1.11]	0.477***[0.34,0.67]	0.594**[0.40,0.88]	0.662 [0.43,1.01]	0.51^***^[0.37,0.69]
**Ethnicity**					
Akan	Ref	Ref	Ref	Ref	Ref
Ga Adangme/ewe	1.00 [0.61,1.22]	1.00 [0.86,1.27]	1.238 [0.99,1.55]	1.265 [0.99,1.61]	1.202 [0.99,1.46]
Mole-dagbani	1.072 [0.73,1.58]	1.206 [0.96,1.52]	1.135 [0.87,1.47]	1.043 [0.78,1.40]	1.082 [0.86,1.36]
Other	1.128 [0.79,1.61]	1.101 [0.89,1.37]	0.903 [0.70,1.16]	0.976 [0.74,1.29]	1.057 [0.85,1.31]
**Employment**					
Not working	Ref	Ref	Ref	Ref	Ref
Managerial	0.90 [0.51,1.56]	0.855 [0.63,1.16]	1.073 [0.76,1.51]	0.953 [0.66,1.39]	0.921 [0.70,1.21]
Clerical /sales	1.00 [0.60,1.01]	0.859* [0.74,1.00]	0.911 [0.77,1.08]	0.755** [0.62,0.91]	0.855^*^[0.74,0.99]
Agricultural	0.81 [0.61,1.08]	1.00 [0.99,1.41]	1.217* [1.01,1.47]	0.959 [0.77,1.19]	1.242^*^[1.04,1.49]
Services	0.325* [0.11,0.92]	0.769 [0.51,1.17]	0.854 [0.52,1.41]	0.421* [0.21,0.86]	0.690 [0.46,1.03]
Manual	0.686* [0.49,0.96]	0.90 [0.78,1.13]	0.954 [0.78,1.17]	0.773* [0.61,0.98]	0.967 [0.81,1.16]
**Religion**					
Christianity	Ref	Ref	Ref	Ref	Ref
Islam	0.813 [0.60,1.11]	0.825* [0.69,0.99]	0.709** [0.58,0.87]	0.772* [0.61,0.98]	0.835 [0.70,1.00]
Traditionalist	2.054** [1.26,3.35]	1.00 [0.91,1.92]	1.033 [0.72,1.47]	1.363 [0.93,1.99]	1.382 [0.92,2.07]
No religion	0.8 [0.48,1.48]	1.092 [0.81,1.47]	0.612** [0.44,0.84]	0.861 [0.59,1.25]	0.903 [0.67,1.23]
**Parity**					
0	Ref	Ref	Ref	Ref	Ref
1 to 3	0.905 [0.65,1.26]	1.431***[1.18,1.73]	0.996 [0.81,1.23]	0.755* [0.59,0.96]	1.108 [0.92,1.33]
4 or more	1.1 [0.69,1.61]	1.846***[1.46,2.34]	1.131 [0.87,1.47]	0.787 [0.58,1.06]	1.334^*^[1.06,1.68]
**Health insurance subscription**					
No	–	Ref	Ref	Ref	Ref
Yes	–	0.542***[0.49,0.61]	0.880* [0.78,0.99]	–	0.59^***^[0.53,0.66]
**Frequency of listening to radio**					
Not at all	Ref	Ref	Ref	Ref	Ref
Less than once a week	1.162 [0.89,1.51]	1.00 [0.77,1.06]	0.927 [0.79,1.10]	0.881 [0.73,1.06]	0.878 [0.75,1.03]
At least once a week	1.047 [0.81,1.36]	0.749***[0.65,0.87]	0.873 [0.74,1.02]	0.793*[0.66,0.95]	0.77^***^[0.66,0.90]
**Frequency of reading newspaper**					
Not at all	–	Ref	Ref		Ref
Less than once a week	–	1.00 [0.76,1.11]	0.911 [0.74,1.13]	–	0.936 [0.78,1.12]
At least once a week	–	0.914 [0.74,1.13]	0.98 [0.77,1.25]	–	0.971 [0.79,1.19]
**Frequency of watching television**					
Not at all	Ref	Ref	Ref	Ref	Ref
Less than once a week	1.383* [1.06,1.80]	0.830* [0.71,0.97]	0.822* [0.69,0.97]	1.125 [0.92,1.37]	0.846^*^[0.72,0.99]
At least once a week	1.00 [0.80,1.38]	0.699***[0.60,0.81]	0.796** [0.68,0.94]	1.132 [0.93,1.37]	0.75^***^[0.64,0.87]
**Household and community level factors**					
**Sex of head of household**					
Male	–	–	Ref	Ref	Ref
Female	–	–	0.915 [0.80,1.05]	0.97 [0.83,1.14]	1.061 [0.94,1.20]
**Wealth**					
Poorest	Ref	Ref	Ref	Ref	Ref
Poorer	1.1 [0.83,1.58]	1.005 [0.83,1.22]	1.008 [0.82,1.23]	1.113 [0.88,1.41]	1.071 [0.88,1.31]
Middle	1.00 [0.72,1.60]	0.863 [0.69,1.09]	1.031 [0.80,1.32]	1.171 [0.87,1.57]	0.944 [0.75,1.19]
Richer	0.758 [0.47,1.21]	0.650** [0.50,0.85]	1.016 [0.76,1.36]	0.958 [0.68,1.35]	0.71^**^[0.54,0.92]
Richest	0.7 [0.40,1.14]	0.399***[0.29,0.54]	0.764 [0.54,1.08]	0.781 [0.53,1.15]	0.47^***^[0.35,0.63]
**Neighbourhood socio-economic status**					
Low	Ref	Ref	Ref	Ref	Ref
Medium	0.812 [0.52,1.27]	1.00 [0.59,1.13]	1.101 [0.75,1.61]	0.837 [0.58,1.21]	0.851 [0.61,1.19]
High	1.041 [0.55,1.97]	0.87 [0.54,1.39]	2.042** [1.20,3.49]	1.416 [0.83,2.40]	1.054 [0.65,1.71]
**Residence**					
Urban	Ref	Ref	Ref	Ref	[1.00,1.00]
Rural	1.00 [0.57,1.42]	0.948 [0.68,1.32]	1.415 [0.97,2.07]	1.024 [0.70,1.49]	0.998 [0.71,1.40]
**Religion**					
Western	Ref	Ref	Ref	Ref	[1.00,1.00]
Central	0.605 [0.35,1.04]	1.00 [0.68,1.59]	0.922 [0.56,1.50]	1.962** [1.19,3.24]	0.95 [0.62,1.45]
Greater Accra	1.00 [0.37,1.18]	0.888 [0.57,1.37]	0.833 [0.50,1.39]	2.463***[1.47,4.11]	0.93 [0.60,1.44]
Volta	0.249***[0.12,0.51]	2.732***[1.73,4.32]	1.214 [0.72,2.04]	1.772* [1.03,3.05]	2.20^**^[1.38,3.53]
Eastern	1.146 [0.68,1.92]	1.661* [1.09,2.52]	2.135** [1.33,3.43]	2.707***[1.65,4.43]	1.70^*^[1.11,2.61]
Ashanti	0.951 [0.57,1.58]	2.041***[1.35,3.08]	1.585 [0.99,2.54]	3.008***[1.85,4.88]	1.75^**^[1.15,2.67]
Brong Ahafo	0.608 [0.35,1.04]	1.0 [0.49,1.15]	0.878 [0.54,1.44]	1.956** [1.18,3.23]	0.776 [0.50,1.19]
Northern	0.90 [0.46,1.66]	2.127** [1.28,3.52]	2.282** [1.29,4.03]	4.740***[2.67,8.43]	2.19^**^[1.30,3.67]
Upper East	0.384**[0.19,0.77]	0.9 [0.53,1.46]	0.510* [0.28,0.92]	1.00 [0.54,1.85]	0.770 [0.46,1.29]
Upper West	1.666 [0.89,3.13]	2.578***[1.55,4.29]	2.672***[1.50,4.75]	1.56 [0.86,2.85]	2.22^**^[1.32,3.74]
N	9370	9370	9370	9370	9370

Exponentiated coefficients; 95% confidence intervals in brackets

*Ref* Reference

Model I is the null model, a baseline model without any determinant variable

Model II = individual level variables

Model III = contextual level variables

Model IV is the final model adjusted for individual and contextual level variables

^*^ *p* < 0.05, ^**^ *p* < 0.01, ^***^ *p* < 0.001

**Table 3:** Multilevel logistic regression of individual and contextual factors associated with barriers to healthcare among women in Ghana (Random effects results)

Barriers	Model I	Model II	Model III	Model IV
**Getting permission to go to hospital**				
N	9370	9370	9370	9370
Community-level variance (SE)	1.0 (0.15)	1.24 (0.92)	0.77 (0.12)	1.03 (0.74)
AIC	4492.093	4453.7	4450.877	4426.39
ICC	0.23	0.22	0.19	0.18
Log-likelihood	− 2244.0	− 2194.9	− 2207.4	− 2165.2
LR Test	213.72 (*p* < 0.001)	175.91 (*p* < 0.001)	146.55 (*p* < 0.001)	127.70 (*p* < 0.001)
**Getting money needed for treatment**				
N	9370	9370	9370	9370
Community-level variance (SE)	1.18 (0.11)	1.20 (0.99)	0.74 (0.07)	0.76 (0.08)
AIC	15,685.7	11,026.2	11,279.4	10,913.5
ICC	0.26	0.23	0.18	1.19
Log-likelihood	− 5808.2	− 5478.1	− 5621.7	− 5405.8
LR Test	1285.46 (*p* < 0.001)	858.01 (*p* < 0.001)	683.13 (*p* < 0.001)	617.98 (*p* < 0.001)
**Distance to health facility**				
N	9370	9370	9370	9370
Community-level variance (SE)	1.76 (0.16)	1.39 (0.14)	0.99 (0.10)	1.18 (0.96)
AIC	9611.4	9531.3	9451.3	9428.922
ICC	0.35	0.30	0.23	0.23
Log-likelihood	− 4803.7	− 4730.631	− 4706.663	− 4662.461
LR Test	1589.25 (*p* < 0.001)	1015.79 (*p* < 0.001)	725.89 (*p* < 0.001)	671.98 (*p* < 0.001)
**Not wanting to go alone**				
N	9370	9370	9370	9370
Community-level variance (SE)	1.08 (0.12)	1.25 (0.99)	0.77 (0.96)	0.76 (0.96)
AIC	7588.2	7481.1	7530.0	7433.4
ICC	0.25	0.23	0.19	0.19
Log-likelihood	− 3792.1	− 3708.5	− 3746.0	− 3667.7
LR Test	618.00 (*p* < 0.001)	451.2 (*p* < 0.001)	346.8 (*p* < 0.001)	304.5 (*p* < 0.001)
**At least one barrier**				
N	9370	9370	9370	9370
Community-level variance (SE)	1.22 (0.11)	1.00 (0.97)	0.82 (0.08)	0.82 (0.08)
AIC	11,610.8	11,147.8	11,309.0	11,051.77
ICC	0.27	0.23	0.200	0.200
Log-likelihood	− 5803.4	− 5538.9	− 5635.5	− 5473.883
LR Test	1317.8 (*p* < 0.001)	907.1 (*p* < 0.001)	760.4 (*p* < 0.001)	703.83 (*p* < 0.001)

*ICC* Intra-Class Correlation, *AIC* Akaike’s Information Criterion, *SE* Standard Error

In terms of marital status, married women had lower odds of facing at least one barrier in healthcare accessibility (AOR = 0.71, CI:0.58–0.87) compared to women who were never married. However, women who were widowed had higher odds in facing barriers to healthcare accessibility (AOR = 1.47, CI: 1.03–2.10). Also married [AOR = 0.60, CI:0.49,0.74] and cohabiting women [AOR = 0.742, CI:0.60,0.92] had lower odds of facing a barrier in terms of getting money needed for treatment compared to never married women. Women with higher level of education had the lowest odds of facing at least one barrier to healthcare accessibility (AOR = 0.51, CI: 0.37–0.69), compared with those with low level of education.

Regarding employment status, those engaged in clerical or sales occupation also had lower odds in facing barriers to healthcare accessibility (AOR = 0.86, CI: 0.74–0.99), compared to the women who were not working. The result also showed that those who were covered by health insurance had lower odds (AOR = 0.59, CI: 0.53–0.66) of barriers to healthcare accessibility, compared to those who were not covered by health insurance. In relation to mass media exposure, specifically, those who listened to radio at least once in a week had lower odds (AOR = 0.77, CI: 0.66–0.90) of barriers to healthcare accessibility, compared to those who did not listen to radio at all. The result also showed that those who watched television at least once a week had lower odds of healthcare accessibility barriers (AOR = 0.75, CI: 0.64–0.87), compared to those who did not watch television at all. Concerning the contextual factors that were considered in the study, the result also showed that women in the richest wealth quantile had lower odds of facing healthcare accessibility barriers, compared to women in the lower wealth quantile (AOR = 0.47, CI: 0.35–0.63). There were also regional variations in barriers to healthcare accessibility. Those in the Volta Region (AOR = 2.20, CI: I.38–3.53) and those in the Upper West Region (AOR = 2.22, CI: 1.32–3.74) had the highest odds of facing barriers to healthcare accessibility, as compared to those in the Western Region. Further details of all the models for each of the barrier to healthcare as well as at least one barrier to healthcare have been presented in the supplementary tables S1, S2 and S3.

## Discussion

This study was undertaken using Ghanaian Demographic and Health Survey data in 2014, and aimed to examine some of the individual as well as the contextual factors associated with the barriers to healthcare for Ghanaian women. The results show that barriers to access to healthcare are widespread among Ghanaian women, with more than half (51%) reporting that there is at least one form of barrier to accessing healthcare. The prevalence of this study is similar to but slightly lower than that reported in South Africa (65%) [22], Rwanda (64%) [9] and Tanzania (65%) [17]. The reason could be as a result of differences in terms of socio-economic development, differences in methodology, as well as certain sociocultural practices in Ghana that predispose women to be subordinated, less empowered, less educated, and also depend on men economically for their needs, which often includes decision-making and financial freedom to access healthcare [23]. Financial accessibility as one of the barriers in this study has been identified as a predominant barrier depriving many women access to healthcare in sub-Saharan Africa [24]. Some other barriers reported in this study include distance to a health facility, not wanting to go alone, and getting permission from spouses or dependents to go to the hospital, and these are in agreement with findings from studies in Nigeria [25] Pakistan [26], and India [27]. This also corroborates with findings from a cross-sectional study in rural Tanzania by Mselle and Kohi [28] which reported that cost of care, distance to a health facility, permission from spouses, not wanting to go alone, and lack of an escort to the health facility were the major barriers facing women in accessing healthcare.

We observed that there was an inverse relationship between age and barriers to healthcare accessibility, where women aged 45–49 had lower odds to report barriers to access healthcare, as compared with women aged 15–19. This finding is similar to findings in Nigeria [25] and Malaysia Lau et al. [29]. In the context of Ghana, the reason for this finding could be that women aged 45–49 are expected to be matured, married, economically active, and financially independent. This puts them in a better position to afford healthcare, coupled with some experiences in managing common morbidity in their localities over time. Conversely, 15–19-year-old women in Ghana are expected to be in school, financially dependent, and dependent on decision-making. As a result, they may not have the financial power and freedom to access healthcare without having to go through someone, thereby creating barriers for them in the process.

In our research, women’s marital status is important for determining the odds of facing barriers to access to healthcare. Compared with married women, widowed women are more likely to face barriers to access to medical care. This finding is consistent with studies conducted in Southern Ethiopia [7], Tanzania [17], Ethiopian Afar Region [30], Montenegro [31] and Malaysia [32]. In contrast, this finding is not important in a study in Japan, where it is reported that marital status does not affect access to medical care [33]. This variation in findings could be as a result of disparities in socioeconomic conditions and socio-cultural practices in each country. In Ghana, certain social and cultural customs deprive widows of full inheritance, social protection and medical care. According to Azah [23], some widowhood ceremonies in Ghana usually prevent women from inheriting their partners’ property, which puts them in a state of extreme poverty, marginalization and unable to afford medical expenses. Previous studies have shown that in many low- and middle-income countries, unfavorable social and cultural practices inhibit women’s access to medical care [34]. On the other hand, married women may benefit from better economic and psychosocial support for their spouses [35].

According to our research, we have also found that compared with low-income people, women in the affluent population and women with higher education are less likely to face healthcare barriers. Similar findings have been reported in previous studies in Ghana [36], Tanzania [17], Uganda [37], Afghanistan [38], Ethiopia [8, 30] and southern Mozambique [39]. The reason here may be that the wealthiest people may be more able to afford the costs associated with access to health care, which is a common challenge faced by poor women in Ghana. Educated women are more likely to engage in high-paying jobs, so they can easily afford medical expenses regardless of the cost and geographic location. Educated women are also more aware of their basic human rights and may have higher health literacy. As a result, they are more likely to overcome any form of barriers to access to health care than their counterparts with lower education and lower health literacy, which is a key factor in determining the utilization rate of health care [40].

Relatedly, the employment status of study participants is significant for determining the likelihood of obstacles in obtaining medical services in Ghana, which is consistent with previous research results by Makmor et al. [41]. Makmor et al. [41] studied the sociodemographic and socioeconomic factors associated with access to public clinics. Sun et al. [42] studied sociodemographic factors related to the ability to obtain health care and revealed similar findings. We found that women in clerical or sales occupations in this study were less likely to face obstacles than women who did not work. This finding could be attributed to financial power, independence, and the high educational level of women who are employed in the clerical and sales sector, as compared to the other women who were employed in the agriculture value chain or reported to be unemployed. Higher education and good jobs give women economic strength and independence, enabling them to afford medical care, thereby overcoming cost, distance, and decision-making barriers [25].

We also found that women with health insurance face fewer barriers to accessing healthcare. This can be discussed in the context of the healthcare utilization model of Anderson and Newman [43], which stipulates that medical insurance subscriptions are a contributing factor in achieving access to healthcare. This finding also supports the findings of previous studies in Ghana [44–46]. Health insurance enables women to afford medical care, freely choose the type of care and choose between medical institutions according to their needs and expectations of care, without worrying about the cost [17]. We further observed that access to mass media showed decreased odds of healthcare accessibility barriers, which confirms previous studies in Ethiopia [47], India [48], Bangladesh [49], and rural Malawi [50]. The reason for this could be that listening to the radio and watching television increases ones’ health literacy, which has been identified as key to healthcare utilization [51].

### Strengths and limitations of the study

This study used nationally representative data to assess the barriers women face in accessing healthcare. There was a high response rate and the study’s methodology followed best practices such as gathering data with experienced data collectors and multi-stage sampling. The findings can, therefore, be generalized to all women in their reproductive ages in Ghana. The study also employed advanced statistical models which accounted for both individual and contextual factors. Despite these, the study design was a cross-sectional one and, therefore, causal interpretation cannot be deduced. Finally, since this was secondary data analysis, we could not account for the effects of the health system and health worker-related factors.

## Conclusion

This study reiterates the fact that both individual and contextual factors are associated with barriers to healthcare accessibility. Specifically, age, marital status, employment, health insurance coverage, frequency of listening to radio, frequency of watching television, wealth status, and region of residence are associated with barriers to healthcare accessibility. In order to achieve the Sustainable Development Goals, it is essential to consider these factors and strengthen existing strategies to alleviate the barriers to health care for Ghanaian women such as strengthening the national health insurance scheme and empowering women economically.

## Supplementary Information

**Additional file 1: Table S1.** Multilevel logistic regression of individual and contextual factors associated with getting permission to go to hospital and getting money needed for treatment. **Table S2.** Multilevel logistic regression of individual and contextual factors associated with distance to health facility and not wanting to go alone to seek healthcare. **Table S3.** Multilevel logistic regression of individual and contextual factors associated with at least one barrier in accessing healthcare among women in Ghana.

## Data Availability

The dataset can be accessed at https:// https://dhsprogram.com/data/dataset/.

## Abbreviations

AOR: Adjusted Odds Ratio
CI: Confidence Interval
GDHS: Ghana Demographic and Health Survey
VIF: Variance Inflation Factor
SDG: Sustainable Development Goal
LMICs: Low- and Middle-Income Countries
WHO: World Health Organisation
GSS: Ghana Statistical Service
LLR: Log-likelihood ratio
SE: Standard Error
ICC: Intra-Class Correlation
LR Test: Likelihood ratio Test
AIC: Akaike’s Information Criterion
