# Supplementary material for: Barriers to accessing healthcare among women in Ghana: a multilevel modelling
Source: BMC Public Health. 2020 Dec 17;20:1916. doi: 10.1186/s12889-020-10017-8 (PMC7745480; doi:10.1186/s12889-020-10017-8)
Supplement: Supplementary file 1 — Additional file 1: Table S1. Multilevel logistic regression of individual and contextual factors associated with getting permission to go to hospital and getting money needed for treatment. Table S2. Multilevel logistic regression of individual and contextual factors associated with distance to health facility and not wanting to go alone to seek healthcare. Table S3. Multilevel logistic regression of individual and contextual factors associated with at least one barrier in accessing healthcare among women in Ghana. [file 12889_2020_10017_MOESM1_ESM.docx]

Supplementary Tables

**Table S1:** Multilevel logistic regression of individual and contextual factors associated with getting permission to go to hospital and getting money needed for treatment

|  | **Permission** | | | | **Money** | | | |
| --- | --- | --- | --- | --- | --- | --- | --- | --- |
| **Variables** | **Model 1** | **Model II**  **AOR [95%CI]** | **Model III**  **AOR [95%CI]** | **Model IV**  **AOR [95%CI]** | **Model 1** | **Model II**  **AOR [95%CI]** | **Model III**  **AOR [95%CI]** | **Model IV**  **AOR [95%CI]** |
| Age |  |  |  |  |  |  |  |  |
| 15-19 |  | Ref |  | Ref |  | Ref |  | Ref |
| 20-24 |  | 0.949[0.70,1.28] |  | 0.941[0.70,1.27] |  | 1.00[0.74,1.06] |  | 1.00[0.73,1.05] |
| 25-29 |  | 0.7[0.50,1.08] |  | 0.7[0.51,1.11] |  | 0.703**[0.56,0.88] |  | 0.727** [0.58,0.91] |
| 30-34 |  | 1[0.54,1.27] |  | 1.00[0.56,1.33] |  | 0.8[0.65,1.05] |  | 0.90[0.69,1.12] |
| 35-39 |  | 0.891[0.57,1.40] |  | 0.939[0.60,1.48] |  | 1.00[0.61,1.02] |  | 1.00[0.65,1.09] |
| 40-44 |  | 0.70[0.40,1.07] |  | 0.70[0.42,1.13] |  | 0.814[0.62,1.07] |  | 0.863[0.66,1.14] |
| 45-49 |  | 1.00[0.38,1.08] |  | 1.00[0.40,1.15] |  | 0.703*[0.53,0.93] |  | 0.745*[0.56,0.99] |
| **Marital status** |  |  |  |  |  |  |  |  |
| Never married |  | Ref |  | Ref |  | Ref |  | Ref |
| Married |  | 0.7[0.50,1.03] |  | 0.70[0.52,1.07] |  | 0.561***[0.46,0.68] |  | 0.604***[0.49,0.74] |
| Cohabitation |  | 1.05[0.72,1.53] |  | 1.055[0.72,1.54] |  | 0.717**[0.58,0.89] |  | 0.742** [0.60,0.92] |
| Widowed |  | 0.697[0.37,1.33] |  | 0.738[0.39,1.41] |  | 1.315[0.93,1.87] |  | 1.378[0.97,1.96] |
| Divorced |  | 1.00[0.42,1.12] |  | 1.0[0.42,1.12] |  | 1.2[0.91,1.51] |  | 1.2[0.91,1.53] |
| **Education** |  |  |  |  |  |  |  |  |
| No education |  | Ref |  | Ref |  | Ref |  | Ref |
| Primary |  | 1.1[0.82,1.44] |  | 1.1[0.83,1.47] |  | 0.987[0.84,1.16] |  | 1.007[0.86,1.19] |
| Secondary |  | 1[0.64,1.12] |  | 1.00[0.67,1.19] |  | 0.742***[0.63,0.87] |  | 0.797**[0.68,0.93] |
| Higher |  | 0.522*[0.28,0.97] |  | 0.588[0.31,1.11] |  | 0.388***[0.28,0.54] |  | 0.477***[0.34,0.67] |
| **Ethnicity** |  |  |  |  |  |  |  |  |
| Akan |  | Ref |  | Ref |  | Ref |  | Ref |
| Ga Adangme/Ewe |  | 0.686*[0.50,0.94] |  | 1.00[0.61,1.22] |  | 1.2[0.99,1.42] |  | 1.00[0.86,1.27] |
| Mole-dagbani |  | 1.184[0.86,1.63] |  | 1.072[0.73,1.58] |  | 1.426***[1.16,1.75] |  | 1.206[0.96,1.52] |
| Other |  | 1.065[0.77,1.47] |  | 1.128[0.79,1.61] |  | 1.212[0.98,1.49] |  | 1.101[0.89,1.37] |
| **Employment** |  |  |  |  |  |  |  |  |
| Not working |  | Ref |  | Ref |  | Ref |  | Ref |
| Managerial |  | 0.9[0.49,1.49] |  | 0.90[0.51,1.56] |  | 0.822[0.61,1.11] |  | 0.855[0.63,1.16] |
| Clerical/sales |  | 0.767*[0.59,0.99] |  | 1.00[0.60,1.01] |  | 0.838*[0.72,0.97] |  | 0.859* [0.74,1.00] |
| Agricultural |  | 0.849[0.64,1.13] |  | 0.81[0.61,1.08] |  | 1.244*[1.05,1.48] |  | 1.00[0.99,1.41] |
| Services |  | 0.316*[0.11,0.90] |  | 0.325* [0.11,0.92] |  | 0.755[0.50,1.15] |  | 0.769[0.51,1.17] |
| Manual |  | 0.692*[0.50,0.97] |  | 0.686* [0.49,0.96] |  | 0.9[0.79,1.13] |  | 0.90[0.78,1.13] |
| **Religion** |  |  |  |  |  |  |  |  |
| Christianity |  | Ref |  | Ref |  | Ref |  | Ref |
| Islam |  | 0.869[0.64,1.18] |  | 0.813[0.60,1.11] |  | 0.852[0.71,1.02] |  | 0.825* [0.69,0.99] |
| Traditional religion |  | 1.999**[1.23,3.25] |  | 2.054** [1.26,3.35] |  | 1[0.96,2.06] |  | 1.00[0.91,1.92] |
| No religion |  | 0.8[0.47,1.44] |  | 0.8[0.48,1.48] |  | 1.134[0.84,1.53] |  | 1.092[0.81,1.47] |
| **Parity** |  |  |  |  |  |  |  |  |
| 0 |  | Ref |  | Ref |  | Ref |  | Ref |
| 1-3 |  | 0.921[0.66,1.28] |  | 0.905[0.65,1.26] |  | 1.493***[1.24,1.80] |  | 1.431***[1.18,1.73] |
| 4 or more |  | 1.1[0.75,1.72] |  | 1.1[0.69,1.61] |  | 2.042***[1.62,2.58] |  | 1.846***[1.46,2.34] |
| **Health Insurance coverage** | | |  |  |  |  |  |  |
| No |  |  |  |  |  | 1.0[1.00,1.00] |  | Ref |
| Yes |  |  |  |  |  | 0.537***[0.48,0.60] |  | 0.542***[0.49,0.61] |
| **Frequency of listening to radio** | | |  |  |  |  |  |  |
| Not at all |  | Ref |  | Ref |  | Ref |  | Ref |
| Less than once a week |  | 1.168[0.90,1.52] |  | 1.162[0.89,1.51] |  | 1.00[0.76,1.03] |  | 1.00[0.77,1.06] |
| At least once a week |  | 1.054[0.81,1.37] |  | 1.047[0.81,1.36] |  | 0.742***[0.64,0.86] |  | 0.749***[0.65,0.87] |
| **Frequency of reading newspaper/magazine** | | |  |  |  |  |  |  |
| Not at all |  |  |  |  |  | Ref |  | Ref |
| Less than once a week |  |  |  |  |  | 1[0.75,1.08] |  | 1.00[0.76,1.11] |
| At least once a week |  |  |  |  |  | 0.843[0.68,1.04] |  | 0.914[0.74,1.13] |
| **Frequency of watching television** | | |  |  |  |  |  |  |
| Not at all |  | Ref |  | Ref |  | Ref |  | Ref |
| Less than once a week |  | 1.304*[1.01,1.69] |  | 1.383* [1.06,1.80] |  | 0.771***[0.66,0.90] |  | 0.830* [0.71,0.97] |
| At least once a week |  | 1[0.76,1.27] |  | 1.00[0.80,1.38] |  | 0.624***[0.54,0.72] |  | 0.699***[0.60,0.81] |
| **Contextual factors** | |  |  |  |  |  |  |  |
| **Sex of household head** | |  |  |  |  |  |  |  |
| Male |  |  |  |  |  |  |  |  |
| Female |  |  |  |  |  |  |  |  |
| **Wealth status** |  |  |  |  |  |  |  |  |
| Poorest |  |  | Ref | Ref |  |  | Ref | Ref |
| Poorer |  |  | 1.1[0.83,1.53] | 1.1[0.83,1.58] |  |  | 0.851[0.71,1.02] | 1.005[0.83,1.22] |
| Middle |  |  | 1[0.69,1.46] | 1.00[0.72,1.60] |  |  | 0.589***[0.48,0.73] | 0.863[0.69,1.09] |
| Richer |  |  | 0.665[0.43,1.02] | 0.758[0.47,1.21] |  |  | 0.331***[0.26,0.42] | 0.650** [0.50,0.85] |
| Richest |  |  | 0.552*[0.34,0.89] | 0.7[0.40,1.14] |  |  | 0.158***[0.12,0.21] | 0.399***[0.29,0.54] |
| **Neighbourhood Socio-Economic Status** | | |  |  |  |  |  |  |
| Low |  |  | Ref | Ref |  |  | Ref | Ref |
| Medium | |  | 0.764[0.49,1.19] | 0.812[0.52,1.27] |  |  | 1.00[0.58,1.09] | 1.01[0.57,1.08] |
| High |  |  | 0.985[0.52,1.85] | 1.041[0.55,1.97] |  |  | 0.965[0.61,1.52] | 0.87[0.54,1.39] |
| **Place of Residence** | |  |  |  |  |  |  |  |
| Rural |  |  | Ref | Ref |  |  | Ref | Ref |
| Urban |  |  | 1[0.61,1.48] | 1.00[0.57,1.42] |  |  | 0.953[0.69,1.32] | 0.948[0.68,1.32] |
| **Region of Residence** | |  |  |  |  |  |  |  |
| Western |  |  | Ref | Ref |  |  | Ref] | Ref |
| Central |  |  | 0.566*[0.33,0.98] | 0.605[0.35,1.04] |  |  | 1[0.71,1.62] | 1.00[0.68,1.59] |
| Greater Accra |  |  | 0.562*[0.32,0.99] | 1.00[0.37,1.18] |  |  | 0.96[0.63,1.46] | 0.888[0.57,1.37] |
| Volta |  |  | 0.226***[0.12,0.44] | 0.249***[0.12,0.51] |  |  | 2.352***[1.54,3.60] | 2.732***[1.73,4.32] |
| Eastern |  |  | 1.02[0.61,1.70] | 1.146[0.68,1.92] |  |  | 1[1.00,2.25] | 1.661* [1.09,2.52] |
| Ashanti |  |  | 0.89[0.53,1.49] | 0.951[0.57,1.58] |  |  | 2.198***[1.47,3.29] | 2.041***[1.35,3.08] |
| Brong Ahafo |  |  | 0.599[0.35,1.03] | 0.608[0.35,1.04] |  |  | 0.648*[0.43,0.98] | 1.0[0.49,1.15] |
| Northern |  |  | 0.8[0.44,1.48] | 0.90[0.46,1.66] |  |  | 1.702*[1.05,2.75] | 2.127** [1.28,3.52] |
| Upper east |  |  | 0.36**[0.19,0.70] | 0.38**[0.19,0.77] |  |  | 0.7[0.44,1.16] | 0.9[0.53,1.46] |
| Upper west |  |  | 1.483[0.83,2.64] | 1.666[0.89,3.13] |  |  | 1.835*[1.14,2.95] | 2.578***[1.55,4.29] |
| **N** | 9370 | 9370 | 9370 | 9370 | 9370 | 9370 | 9370 | 9370 |

Exponentiated coefficients; 95% confidence intervals in brackets

^*^ *p* < 0.05, ^**^ *p* < 0.01, ^***^ *p* < 0.001

Ref=Reference

Model I is the null model, a baseline model without any determinant variable

Model II = individual level variables

Model III = contextual level variables

Model IV is the final model adjusted for individual and contextual level variables

**Table S2:** Multilevel logistic regression of individual and contextual factors associated with distance to health facility and not wanting to go alone to seek healthcare

|  | **Distance** | | | | **Alone** | | | |
| --- | --- | --- | --- | --- | --- | --- | --- | --- |
| **Variables** | **Model 1** | **Model II**  **AOR [95%CI]** | **Model III**  **AOR [95%CI]** | **Model IV**  **AOR [95%CI]** | **Model 1** | **Model II**  **AOR [95%CI]** | **Model III**  **AOR [95%CI]** | **Model IV**  **AOR [95%CI]** |
| Age |  |  |  |  |  |  |  |  |
| 15-19 |  | 1[1.00,1.00] |  | Ref |  | 1[1.00,1.00] |  | Ref |
| 20-24 |  | 0.898[0.73,1.10] |  | 0.915[0.75,1.12] |  | 0.614***[0.49,0.77] |  | 0.613***[0.49,0.77] |
| 25-29 |  | 0.781*[0.61,1.00] |  | 0.823[0.64,1.05] |  | 0.580***[0.44,0.76] |  | 0.592***[0.45,0.78] |
| 30-34 |  | 0.819[0.62,1.07] |  | 0.887[0.67,1.17] |  | 0.591***[0.44,0.80] |  | 0.614**[0.45,0.84] |
| 35-39 |  | 0.798[0.60,1.06] |  | 0.877[0.66,1.17] |  | 0.548***[0.40,0.76] |  | 0.576**[0.41,0.80] |
| 40-44 |  | 0.807[0.59,1.09] |  | 0.893[0.66,1.21] |  | 0.515***[0.36,0.73] |  | 0.547***[0.38,0.78] |
| 45-49 |  | 0.724*[0.53,0.99] |  | 0.805[0.59,1.11] |  | 0.525***[0.37,0.75] |  | 0.555** [0.39,0.80] |
| **Marital status** |  |  |  |  |  |  |  |  |
| Never married |  | Ref |  | Ref |  | Ref |  | Ref |
| Married |  | 0.879[0.70,1.10] |  | 0.854[0.67,1.08] |  | 1.025[0.79,1.33] |  | 1.05[0.80,1.38] |
| Cohabitation |  | 0.901[0.71,1.15] |  | 0.866[0.67,1.11] |  | 0.95[0.72,1.26] |  | 0.937[0.70,1.25] |
| Widowed |  | 0.951[0.65,1.40] |  | 0.976[0.66,1.43] |  | 1.449[0.94,2.24] |  | 1.498[0.97,2.32] |
| Divorced |  | 0.978[0.73,1.31] |  | 0.996[0.74,1.34] |  | 0.996[0.70,1.42] |  | 1.016[0.71,1.45] |
| **Education** |  |  |  |  |  |  |  |  |
| No education |  | Ref |  | Ref |  | Ref |  | Ref |
| Primary |  | 0.879[0.74,1.05] |  | 0.914[0.77,1.09] |  | 0.794*[0.64,0.98] |  | 0.835[0.68,1.03] |
| Secondary |  | 0.843[0.71,1.00] |  | 0.908[0.76,1.08] |  | 0.773*[0.63,0.95] |  | 0.835[0.68,1.03] |
| Higher |  | 0.515***[0.35,0.76] |  | 0.594**[0.40,0.88] |  | 0.575**[0.38,0.87] |  | 0.662[0.43,1.01] |
| **Ethnicity** |  |  |  |  |  |  |  |  |
| Akan |  | 1[1.00,1.00] |  | Ref |  | 1[1.00,1.00] |  | Ref |
| Ga Adangme/Ewe |  | 1.271*[1.03,1.56] |  | 1.238[0.99,1.55] |  | 1.315*[1.05,1.64] |  | 1.265[0.99,1.61] |
| Mole-dagbani |  | 1.411**[1.12,1.78] |  | 1.135[0.87,1.47] |  | 1.093[0.85,1.41] |  | 1.043[0.78,1.40] |
| Other |  | 1.046[0.82,1.33] |  | 0.903[0.70,1.16] |  | 1.068[0.83,1.38] |  | 0.976[0.74,1.29] |
| **Employment** |  |  |  |  |  |  |  |  |
| Not working |  | Ref |  | Ref |  | Ref |  | Ref |
| Managerial |  | 1.031[0.73,1.45] |  | 1.073[0.76,1.51] |  | 0.926[0.64,1.34] |  | 0.953[0.66,1.39] |
| Clerical/sales |  | 0.872[0.74,1.03] |  | 0.911[0.77,1.08] |  | 0.760**[0.63,0.92] |  | 0.755** [0.62,0.91] |
| Agricultural |  | 1.309**[1.09,1.58] |  | 1.217* [1.01,1.47] |  | 1.023[0.83,1.26] |  | 0.959[0.77,1.19] |
| Services |  | 0.809[0.49,1.34] |  | 0.854[0.52,1.41] |  | 0.419*[0.21,0.86] |  | 0.421* [0.21,0.86] |
| Manual |  | 0.9[0.77,1.16] |  | 0.954[0.78,1.17] |  | 0.777*[0.61,0.99] |  | 0.773* [0.61,0.98] |
| **Religion** |  |  |  |  |  |  |  |  |
| Christianity |  | Ref |  | Ref |  | Ref |  | Ref |
| Islam |  | 0.732**[0.59,0.90] |  | 0.709** [0.58,0.87] |  | 0.863[0.69,1.09] |  | 0.772* [0.61,0.98] |
| Traditional religion |  | 1.075[0.75,1.54] |  | 1.033[0.72,1.47] |  | 1.432[0.98,2.09] |  | 1.363[0.93,1.99] |
| No religion |  | 0.616**[0.45,0.85] |  | 0.612** [0.44,0.84] |  | 0.878[0.61,1.27] |  | 0.861[0.59,1.25] |
| **Parity** |  |  |  |  |  |  |  |  |
| 0 |  | Ref |  | Ref |  | Ref |  | Ref |
| 1-3 |  | 1.006[0.82,1.24] |  | 0.996[0.81,1.23] |  | 0.771*[0.61,0.98] |  | 0.755* [0.59,0.96] |
| 4 or more |  | 1.188[0.92,1.54] |  | 1.131[0.87,1.47] |  | 0.824[0.61,1.11] |  | 0.787[0.58,1.06] |
| **Health Insurance coverage** | | |  |  |  |  |  |  |
| No |  | Ref |  | Ref |  |  |  |  |
| Yes |  | 0.897[0.79,1.01] |  | 0.880* [0.78,0.99] |  |  |  |  |
| **Frequency of listening to radio** | | |  |  |  |  |  |  |
| Not at all |  | Ref |  | Ref |  | Ref |  | Ref |
| Less than once a week |  | 0.918[0.78,1.08] |  | 0.927[0.79,1.10] |  | 0.851[0.71,1.02] |  | 0.881[0.73,1.06] |
| At least once a week |  | 0.879[0.75,1.03] |  | 0.873[0.74,1.02] |  | 0.776**[0.65,0.93] |  | 0.793*[0.66,0.95] |
| **Frequency of reading newspaper/magazine** | | |  |  |  |  |  |  |
| Not at all |  | Ref |  | Ref |  |  |  |  |
| Less than once a week |  | 0.878[0.71,1.09] |  | 0.911[0.74,1.13] |  |  |  |  |
| At least once a week |  | 0.923[0.72,1.18] |  | 0.98[0.77,1.25] |  |  |  |  |
| **Frequency of watching television** | | |  |  |  |  |  |  |
| Not at all |  | Ref |  | Ref |  | Ref |  | Ref |
| Less than once a week |  | 0.762**[0.64,0.90] |  | 0.822* [0.69,0.97] |  | 1.067[0.88,1.30] |  | 1.125[0.92,1.37] |
| At least once a week |  | 0.710***[0.61,0.83] |  | 0.796** [0.68,0.94] |  | 1.059[0.88,1.27] |  | 1.132[0.93,1.37] |
| **Contextual factors** |  |  |  |  |  |  |  |  |
| **Sex of household head** | |  |  |  |  |  |  |  |
| Male |  |  | Ref | Ref |  |  | Ref | Ref |
| Female |  |  | 0.972[0.86,1.10] | 0.915[0.80,1.05] |  |  | 1.03[0.90,1.18] | 0.97[0.83,1.14] |
| **Wealth status** |  |  |  |  |  |  |  |  |
| Poorest |  |  | Ref | Ref |  |  | Ref | Ref |
| Poorer |  |  | 0.902[0.74,1.10] | 1.008[0.82,1.23] |  |  | 1.029[0.82,1.29] | 1.113[0.88,1.41] |
| Middle |  |  | 0.815[0.64,1.03] | 1.031[0.80,1.32] |  |  | 1.002[0.76,1.32] | 1.171[0.87,1.57] |
| Richer |  |  | 0.719*[0.55,0.94] | 1.016[0.76,1.36] |  |  | 0.81[0.59,1.11] | 0.958[0.68,1.35] |
| Richest |  |  | 0.502***[0.37,0.69] | 0.764[0.54,1.08] |  |  | 0.662*[0.46,0.94] | 0.781[0.53,1.15] |
| **Neighbourhood Socio-Economic Status** | | |  |  |  |  |  |  |
| Low |  |  | Ref | Ref |  |  | Ref | Ref |
| Medium | |  | 1.032[0.71,1.51] | 1.101[0.75,1.61] |  |  | 0.792[0.55,1.15] | .836[0.57,1.21] |
| High |  |  | 2.016*[1.18,3.44] | 2.042** [1.20,3.49] |  |  | 1.333[0.79,2.25] | 1.416[0.83,2.40] |
| **Place of Residence** |  |  |  |  |  |  |  |  |
| Rural |  |  | 1[1.00,1.00] | Ref |  |  | 1[1.00,1.00] | Ref |
| Urban |  |  | 1.57*[1.07,2.29] | 1.415[0.97,2.07] |  |  | 1.098[0.76,1.59] | 1.024[0.70,1.49] |
| **Region of Residence** |  |  |  |  |  |  |  |  |
| Western |  |  | Ref | Ref |  |  | Ref | Ref |
| Central |  |  | 0.917[0.56,1.50] | 0.922[0.56,1.50] |  |  | 1.910*[1.16,3.15] | 1.962** [1.19,3.24] |
| Greater Accra |  |  | 0.896[0.54,1.49] | 0.833[0.50,1.39] |  |  | 2.500***[1.51,4.13] | 2.463***[1.47,4.11] |
| Volta |  |  | 1.326[0.81,2.18] | 1.214[0.72,2.04] |  |  | 2.078**[1.25,3.47] | 1.772* [1.03,3.05] |
| Eastern |  |  | 2.20**[1.37,3.54] | 2.135** [1.33,3.43] |  |  | 2.700***[1.66,4.40] | 2.707***[1.65,4.43] |
| Ashanti |  |  | 1.60[0.99,2.56] | 1.585[0.99,2.54] |  |  | 2.805***[1.73,4.55] | 3.008***[1.85,4.88] |
| Brong Ahafo |  |  | 0.822[0.50,1.35] | 0.878[0.54,1.44] |  |  | 1.867*[1.13,3.08] | 1.956** [1.18,3.23] |
| Northern |  |  | 1.965*[1.13,3.42] | 2.282** [1.29,4.03] |  |  | 4.493***[2.59,7.79] | 4.740***[2.67,8.43] |
| Upper east |  |  | 0.477*[0.27,0.84] | 0.510* [0.28,0.92] |  |  | 0.995[0.56,1.78] | 1.00[0.54,1.85] |
| Upper west |  |  | 2.412**[1.39,4.18] | 2.672***[1.50,4.75] |  |  | 1.532[0.87,2.71] | 1.56[0.86,2.85] |
| **N** | 9370 | 9370 | 9370 | 9370 | 9370 | 9370 | 9370 | 9370 |

Exponentiated coefficients; 95% confidence intervals in brackets

^*^ *p* < 0.05, ^**^ *p* < 0.01, ^***^ *p* < 0.001

Ref=Reference

Model I is the null model, a baseline model without any determinant variable

Model II = individual level variables

Model III = contextual level variables

Model IV is the final model adjusted for individual and household/community level variables

**Table S3:** Multilevel logistic regression of individual and contextual factors associated with at least one barrier in accessing healthcare among women in Ghana

| **Variables** | **Model 1** | **Model II**  **AOR [95%CI]** | **Model III**  **AOR [95%CI]** | **Model IV**  **AOR [95%CI]** |
| --- | --- | --- | --- | --- |
| Age |  |  |  |  |
| 15-19 |  | Ref |  | Ref |
| 20-24 |  | 0.833^*^[0.70,1.00] |  | 0.820^*^[0.68,0.98] |
| 25-29 |  | 0.66^***^[0.53,0.82] |  | 0.67^***^[0.54,0.83] |
| 30-34 |  | 0.707^**^[0.56,0.90] |  | 0.729^*^[0.57,0.93] |
| 35-39 |  | 0.690^**^[0.53,0.89] |  | 0.716^*^[0.55,0.93] |
| 40-44 |  | 0.731^*^[0.56,0.96] |  | 0.750^*^[0.57,0.99] |
| 45-49 |  | 0.630^**^[0.48,0.84] |  | 0.65^**^[0.49,0.86] |
| **Marital status** |  |  |  |  |
| Never married |  | Ref |  | Ref |
| Married |  | 0.64^***^[0.52,0.77] |  | 0.71^**^[0.58,0.87] |
| Cohabitation |  | 0.832[0.67,1.03] |  | 0.882[0.71,1.10] |
| Widowed |  | 1.429^*^[1.01,2.04] |  | 1.469^*^[1.03,2.10] |
| Divorced |  | 1.114[0.86,1.44] |  | 1.116[0.86,1.44] |
| **Education** |  |  |  |  |
| No education |  | Ref |  | Ref |
| Primary |  | 0.897[0.76,1.06] |  | 0.903[0.76,1.07] |
| Secondary |  | 0.71^***^[0.61,0.83] |  | 0.75^***^[0.64,0.89] |
| Higher |  | 0.43^***^[0.32,0.58] |  | 0.51^***^[0.37,0.69] |
| **Ethnicity** |  |  |  |  |
| Akan |  | Ref |  | Ref |
| Ga Adangme/Ewe |  | 1.245^*^[1.03,1.50] |  | 1.202[0.99,1.46] |
| Mole-dagbani |  | 1.169[0.94,1.45] |  | 1.082[0.86,1.36] |
| Other |  | 1.089[0.88,1.34] |  | 1.057[0.85,1.31] |
| **Employment** |  |  |  |  |
| Not working |  | Ref |  | Ref |
| Managerial |  | 0.895[0.68,1.18] |  | 0.921[0.70,1.21] |
| Clerical/sales |  | 0.852^*^[0.74,0.99] |  | 0.855^*^[0.74,0.99] |
| Agricultural |  | 1.284^**^[1.07,1.53] |  | 1.242^*^[1.04,1.49] |
| Services |  | 0.697[0.47,1.04] |  | 0.690[0.46,1.03] |
| Manual |  | 0.977[0.82,1.17] |  | 0.967[0.81,1.16] |
| **Religion** |  |  |  |  |
| Christianity |  | Ref |  | Ref |
| Islam |  | 0.872[0.73,1.04] |  | 0.835[0.70,1.00] |
| Traditional religion |  | 1.401[0.93,2.10] |  | 1.382[0.92,2.07] |
| No religion |  | 0.913[0.67,1.24] |  | 0.903[0.67,1.23] |
| **Parity** |  |  |  |  |
| 0 |  | Ref |  | Ref |
| 1-3 |  | 1.159[0.97,1.39] |  | 1.108[0.92,1.33] |
| 4 or more |  | 1.462^**^[1.16,1.84] |  | 1.334^*^[1.06,1.68] |
| **Health Insurance coverage** | | |  |  |
| No |  | Ref |  | Ref |
| Yes |  | 0.58^***^[0.52,0.64] |  | 0.59^***^[0.53,0.66] |
| **Frequency of listening to radio** | | |  |  |
| Not at all |  | Ref |  | Ref |
| Less than once a week |  | 0.866[0.74,1.01] |  | 0.878[0.75,1.03] |
| At least once a week |  | 0.77^***^[0.66,0.89] |  | 0.77^***^[0.66,0.90] |
| **Frequency of reading newspaper/magazine** | | |  |  |
| Not at all |  | Ref |  | Ref |
| Less than once a week |  | 0.912[0.76,1.09] |  | 0.936[0.78,1.12] |
| At least once a week |  | 0.919[0.75,1.12] |  | 0.971[0.79,1.19] |
| **Frequency of watching television** | | |  |  |
| Not at all |  | Ref |  | [Ref |
| Less than once a week |  | 0.803^**^[0.69,0.94] |  | 0.846^*^[0.72,0.99] |
| At least once a week |  | 0.69^***^[0.59,0.80] |  | 0.75^***^[0.64,0.87] |
| **Contextual factors** |  |  |  |  |
| **Sex of household head** | |  |  |  |
| Male |  |  | Ref | Ref |
| Female |  |  | 1.23^***^[1.11,1.37] | 1.061[0.94,1.20] |
| **Wealth status** |  |  |  |  |
| Poorest |  |  | Ref | Ref |
| Poorer |  |  | 0.904[0.75,1.10] | 1.071[0.88,1.31] |
| Middle |  |  | 0.65^***^[0.52,0.80] | 0.944[0.75,1.19] |
| Richer |  |  | 0.39^***^[0.30,0.49] | 0.71^**^[0.54,0.92] |
| Richest |  |  | 0.21^***^[0.16,0.28] | 0.47^***^[0.35,0.63] |
| **Neighbourhood Socio-Economic Status** | |  |  |  |
| Low |  |  | Ref | Ref |
| Medium | |  | 0.834[0.60,1.16] | 0.851[0.61,1.19] |
| High |  |  | 1.184[0.74,1.90] | 1.054[0.65,1.71] |
| **Place of Residence** |  |  |  |  |
| Rural |  |  | Ref | Ref |
| Urban |  |  | 1.033[0.74,1.44] | 0.998[0.71,1.40] |
| **Region of Residence** |  |  |  |  |
| Western |  |  | Ref | Ref |
| Central |  |  | 0.967[0.63,1.48] | 0.95[0.62,1.45] |
| Greater Accra |  |  | 1.038[0.68,1.59] | 0.93[0.60,1.44] |
| Volta |  |  | 2.17^***^[1.39,3.38] | 2.20^**^[1.38,3.53] |
| Eastern |  |  | 1.607^*^[1.05,2.45] | 1.70^*^[1.11,2.61] |
| Ashanti |  |  | 1.843^**^[1.21,2.80] | 1.75^**^[1.15,2.67] |
| Brong Ahafo |  |  | 0.683[0.44,1.05] | 0.776[0.50,1.19] |
| Northern |  |  | 1.821^*^[1.10,3.00] | 2.19^**^[1.30,3.67] |
| Upper east |  |  | 0.628[0.38,1.03] | 0.770[0.46,1.29] |
| Upper west |  |  | 1.649^*^[1.00,2.71] | 2.22^**^[1.32,3.74] |
| **N** | 9370 | 9370 | 9370 | 9370 |

**Source: 2014 GDHS**

Exponentiated coefficients; 95% confidence intervals in brackets

^*^ *p* < 0.05, ^**^ *p* < 0.01, ^***^ *p* < 0.001

Ref=Reference

Model I is the null model, a baseline model without any determinant variable

Model II = individual level variables

Model III = contextual level variables

Model IV is the final model adjusted for individual and Contextual level variables
